# Predicting implicit concept embeddings for singular relationship discovery replication of closed literature-based discovery

**DOI:** 10.3389/frma.2025.1509502

**Published:** 2025-03-05

**Authors:** Clint Cuffy, Bridget T. McInnes

**Affiliations:** Natural Language Processing Lab, Department of Computer Science, Virginia Commonwealth University, Richmond, VA, United States

**Keywords:** natural language processing, semantic similarity and relatedness, distributional similarity, literature-based discovery, neural networks, deep learning, knowledge discovery

## Abstract

**Objective:**

Literature-based Discovery (LBD) identifies new knowledge by leveraging existing literature. It exploits interconnecting implicit relationships to build bridges between isolated sets of non-interacting literatures. It has been used to facilitate drug repurposing, new drug discovery, and study adverse event reactions. Within the last decade, LBD systems have transitioned from using statistical methods to exploring deep learning (DL) to analyze semantic spaces between non-interacting literatures. Recent works explore knowledge graphs (KG) to represent explicit relationships. These works envision LBD as a knowledge graph completion (KGC) task and use DL to generate implicit relationships. However, these systems require the researcher to have domain-expert knowledge when submitting relevant queries for novel hypothesis discovery.

**Methods:**

Our method explores a novel approach to identify all implicit hypotheses given the researcher's search query and expedites the knowledge discovery process. We revise the KGC task as the task of predicting interconnecting vertex embeddings within the graph. We train our model using a similarity learning objective and compare our model's predictions against all known vertices within the graph to determine the likelihood of an implicit relationship (i.e., connecting edge). We also explore three approaches to represent edge connections between vertices within the KG: average, concatenation, and Hadamard. Lastly, we explore an approach to induce inductive biases and expedite model convergence (i.e., input representation scaling).

**Results:**

We evaluate our method by replicating five known discoveries within the Hallmark of Cancer (HOC) datasets and compare our method to two existing works. Our results show no significant difference in reported ranks and model convergence rate when comparing scaling our input representations and not using this method. Comparing our method to previous works, we found our method achieves optimal performance on two of five datasets and achieves comparable performance on the remaining datasets. We further analyze our results using statistical significance testing to demonstrate the efficacy of our method.

**Conclusion:**

We found our similarity-based learning objective predicts linking vertex embeddings for single relationship closed discovery replication. Our method also provides a ranked list of linking vertices between a set of inputs. This approach reduces researcher burden and allows further exploration of generated hypotheses.

## 1 Introduction

As research is published in scientific repositories, the subject content of these publications becomes increasingly discretized with each passing year. This creates distant collections of isolated literatures which conceals thought-provoking details and hidden relationships between subjects of interest. Literature-based Discovery (LBD) is the systematic process of creating interconnecting paths between these collections of literatures. It accomplishes this by identifying hidden connections between definitive relationships to unveil latent mechanistic connections within literature and discover novel knowledge.

LBD was initially developed by Swanson (1986) when identifying potential treatments for Raynaud's Disease and later formalized into a generalizable method for novel discovery proposal (i.e., the *A-B-C* paradigm). Since then, researchers have looked a means of automating the process (Luo et al., 2018; Thilakaratne et al., 2018) and applied their methods to various applications (e.g., knowledge discovery, replication, and visualization) across many domains (e.g., general, IoT, and biomedical publications). Existing works in these application areas include applying LBD systems to study the long-term effects of bullying (Hasan, 2019), discovering links between food security and the IoT (Mejía and Kajikawa, 2021), studying climate change (Aamot, 2014), expediting the development of efficient water purification systems (Kostoff et al., 2008), and accelerating development in developing countries (Gordon and Awad, 2008). While LBD remains generalizable across any domain, it requires considerable resources (time and financial) to curate data for novel knowledge discovery and evaluate system performance. Due to the prevalence of existing biomedical data sources, ontologies, and databases such as UMLS (Bodenreider, 2004), PubMed (PubMed, 1996), and SemMedDB (Kilicoglu et al., 2012), our desire as researchers to study the interactions between living organisms and discover novel actionable insights to improve the quality of life (i.e., disease treatment and improving patient healthcare outcomes), the majority of studies apply LBD methods within the biomedical domain. These studies include the following use cases: development of new drugs (i.e., new drug discovery) (Zhao et al., 2019; Rindflesch et al., 2018; Sang et al., 2018b; McCoy et al., 2021), repurposing of existing drugs for symptom management and disease treatment (e.g., drug repurposing or repositioning) (Rastegar-Mojarad et al., 2015; Brown and Patel, 2016; Yang et al., 2016; Daowd et al., 2022; Tropmann-Frick and Schreier, 2022; Sang et al., 2018b; McCoy et al., 2021), and studying adverse side-effects of existing drugs (e.g., adverse drug reactions) (Shang et al., 2014; Hristovski et al., 2016; Rastegar-Mojarad et al., 2016; Rindflesch et al., 2018). LBD methods have been successfully applied to all aforementioned case studies while alleviating the large associated resource requirements (i.e., time and monetary expense).

At its inception, LBD was greatly influenced by statistical modeling methods to identify new knowledge. However, within the last decade, deep learning (DL) has facilitated the exploration of alternative methods to perform LBD. Current trends include the use of graph theory methods to identify fruitful connections for knowledge discovery. In this setting, knowledge graphs (KG) are constructed from explicit *A-B-C* relationships which are extracted from queried documents. To stimulate novel relationships, models are trained to predict links between graphed elements (i.e., *link prediction*). These methods accept term (or concept) triplets and predict a score indicating the likelihood of connecting edges between the elements within the query. While this method has been shown to produce plausible connections between vertices within the graph, it requires researchers to obtain domain-specific knowledge for crafting and submitting relevant queries to generate or test hypotheses. This high barrier to entry also reduces the researcher's inclination to further explore proposed connections by the system.

In this work, we present a novel approach for knowledge graph completion tailored to closed literature-based discovery (LBD). Our model leverages Unified Medical Language System (UMLS) concepts to represent terms within a knowledge graph (KG), focusing on node prediction rather than traditional classification objectives. Specifically, we train our model to predict the embedding of a concept A-B-C relationship, offering a new perspective on embedding-based KGC. To enhance the representation of relationships between concepts, we explore three distinct approaches: 1) averaging, 2) concatenation, and 3) computing the Hadamard product of concept embeddings. Our method demonstrates that these approaches effectively capture relationships within the Hallmarks of Cancer (HOC) dataset, revealing hidden connections that traditional methods might overlook. Significantly, our method alleviates the amount of time necessary to generate novel discoveries by automating the discovery of plausible (implicit) relationships between concepts. By computing connecting edges to all unique concepts, we reduce the manual effort required to identify and validate relationships, thus making the knowledge discovery process more accessible and efficient to the casual researcher as well as domain experts. First, we provide background information and related works in Sections 2.1 *(Background)* and 2.2 *(Related work)*. Next, we discuss the data and our methodology in Sections 3 *(Data)* and 4 *(Materials and methods)*. Lastly, we show our results, provide discussion, and address areas of future work in Sections 6 *(Results)*, 7.1 *(Conclusion)*, and 7.2 *(Limitations and future work)*.

## 2 Background and related work

### 2.1 Background

LBD is a systematic process that seeks to identify implicit relationships within existing literature. This process was derived by Swanson (1986) when identifying the relationship between fish oil and Raynaud's disease (i.e., $Fish\;oil\overset{treats}{\to }Raynaud^{\prime }s\;Disease$). The method used to achieve this is the *A-B-C* paradigm. In this paradigm, explicit *A* → *B* and *B* → *C* relationships are leveraged to identify hidden mechanistic connections between terms (i.e., novel *A* → *C* relationships). These identified relationships are used to explain correlations between terms or identify new knowledge by hypothesizing potential connections of interest (i.e., closed vs open discovery).

**Figure 1:**
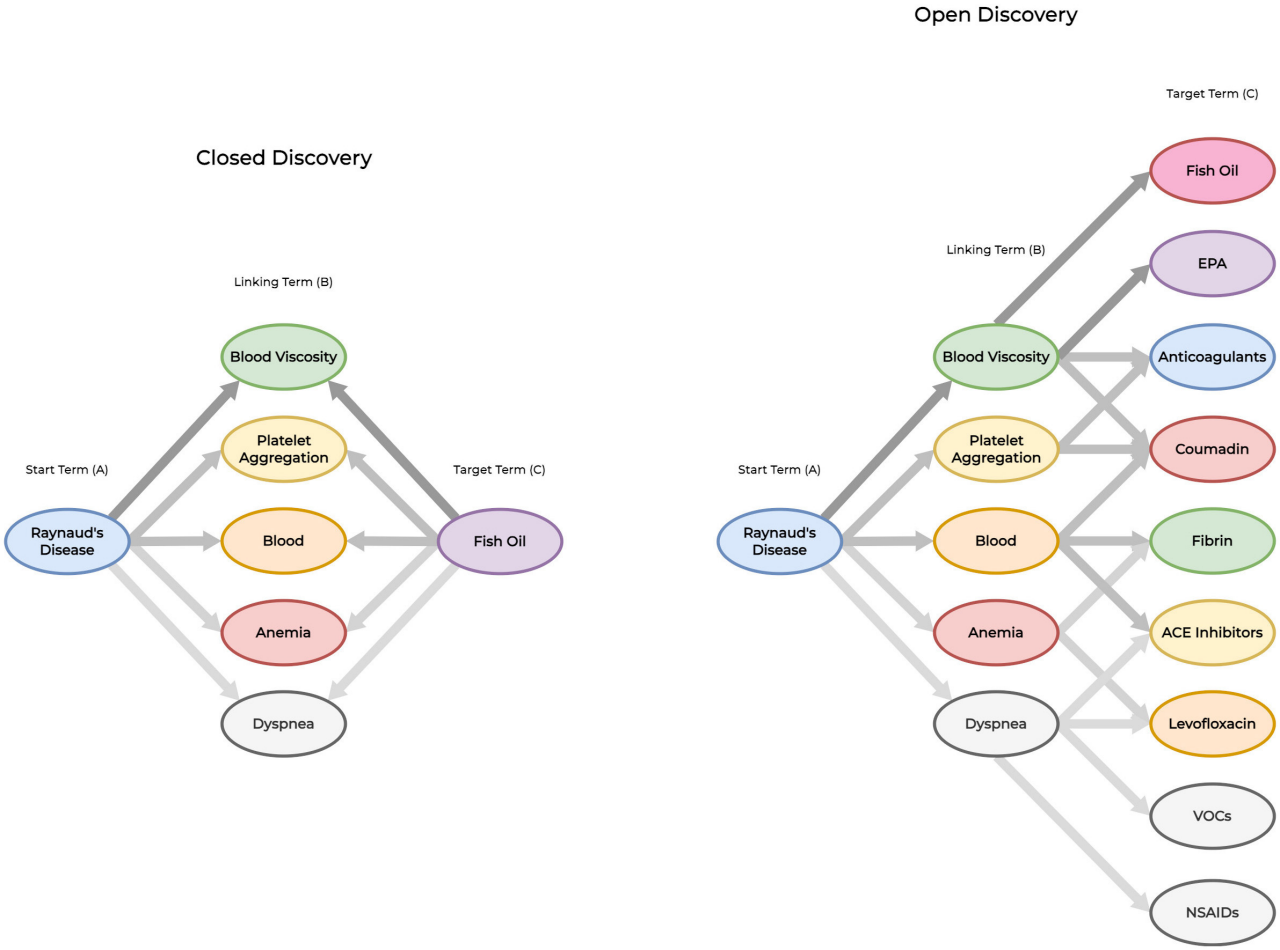
Closed vs. open discovery paradigms. To demonstrate the differences between each approach, we provide an example of an LBD system identifying implicit relationships using both discovery paradigms. Closed discovery captures several *linking terms* to identify potential relationships between the *starting term* (Raynaud's Disease) and *target term* (Fish oil). Open discovery derives new knowledge by providing a *starting term* (Raynaud's Disease) to reveal the implicit relationship *Raynaud's disease-blood viscosity-fish oil* through multiple *linking* and *target* terms. This approach is not limited to identifying a single implicit relationship. We use darker levels of edge contrast between terms to signify stronger relationships between vertices for replicating Swanson (1986)'s first literature-based discovery.

Shown in Figure 1, Swanson (1986) developed two approaches for amplifying implicit features within the knowledge discovery process: closed and open discovery. Each approach utilizes the *A-B-C* paradigm as a basis for identifying relationships. However, there are distinct differences between each approach. In open discovery, researchers are required to submit a starting *(A)* query into the system. Next, the system identifies one or more linking *(B)* terms that are associated with the *A* term. Finally, for each *B* term, the system identifies a set of potential target *C* terms along with confidence (or likelihood) scores for each identified relationship. Closed discovery requires the researcher to submit two elements within their query: the starting *(A)* and target *(C)* terms. Next, the system identifies all relevant novel connections between the query terms as a set of linking *(B)* terms. For either approach, the resulting set of identified terms are ranked by their confidence (or likelihood) scores, and presented to the user for further analysis. Between both approaches, open discovery is often described as an approach used to generate new hypotheses while closed discovery is used to test (or explain) hypotheses.

### 2.2 Related work

Since its inception numerous methods to facilitate relationship exploration among terms (or concepts), and discover new knowledge have been developed. Early works combined term co-occurrence, statistical (Swanson, 1988, 1986; Smalheiser and Swanson, 1998; Smalheiser et al., 2009), and association measures (Hristovski et al., 2005; Hu et al., 2010) to unearth implicit relationships. Limitations of these methods include addressing term lexical variations, grammatical inconsistencies, and spurious terms. With the advent of DL, LBD works addressed the limitations of previous methods by shifting toward leveraging distributed semantics across documents to learn embedded representations for terms, and exploit this semantic information to unearth novel knowledge. Recent works include the use of knowledge graphs to represent connections of interest as relational data. In this setting, terms of interest are represented using Unified Medical Language System (UMLS) (Bodenreider, 2004) concept unique identifiers (CUIs) or Medical Subject Heading (MeSH) (Lipscomb, 2000) terms. To represent relationships, two common approaches are used: (1) co-occurrence relationships, and (2) extracting relationships from relational databases. For either approach, relationships are represented as vertex triplets with connecting edges. Pyysalo et al. (2018), Crichton et al. (2020), Ding and Jin (2021), and Škrlj et al. (2021) use co-occurrence relationship triplets where terms (or concepts) are vertices and the connecting edges between vertices represent co-occurring vertices at the sentence or document-level (e.g., *Fish oil* <>*Blood viscosity* <>*Raynaud*′*s Disease*). In contrast, Preiss and Stevenson (2018), Sang et al. (2018a,b), Sang et al. (2019), Zhang et al. (2021), and Daowd et al. (2022) use relational databases such as the Semantic Medline Database (SemMedDB) (Kilicoglu et al., 2012) which describes relationships as *subject-predicate-object* triples where the *subject* and *object* elements are vertices (UMLS concepts) and the *predicate* are terms which represents the edge bridging the two concepts (e.g., $Fish\;oil\to TREATS\to Raynaud^{\prime }s\;Disease$). Each approach provides distinct advantages. However, careful considerations must be made when selecting the appropriate preprocessing approach. Co-occurrence-based approaches generate undirected graphs where the relationship between connected vertices is bidirectional. In comparison, relational databases generate directed graphs where the relationships are uni-directional.

#### 2.2.1 Knowledge graph completion

A recent trend in the LBD community includes the utilization of knowledge graph completion (KGC) methods to perform LBD. This includes developing systems to identify new (implicit) vertices connecting to an edge (i.e., *node prediction*) or edges between vertices (i.e., *link prediction*). Knowledge graphs constructed from co-occurrence and relational databases use the standard definition of a graph: *G* = (*V, E*), where *V* represents vertices (or nodes) and *E* represents edges (or links). For either KGC approach, relationships are represented in the graph as *G* = (*h, r, t*) where each variable differs depending on the preprocessing approach chosen. Using concept co-occurrence, all variables are vertices and represented as terms (or concepts) (e.g., *Fish oil* <>*Blood viscosity* <>*Raynaud*′*s Disease*). (*h, r, t*) triplets are co-occurring. Therefore, edges exist between them within the graph. Relational databases represent these relationship variables differently with *h* and *t* as concept vertices, and *r* as the edge (predicate) which describes the relationship between them (e.g., $Fish\;oil\to TREATS\to Raynaud^{\prime }s\;Disease$). Two common approaches are used to perform *link* or *node prediction*: 1) we submit (*h, r, t*) triplets and query the system to identify plausible links between all triplet nodes (i.e., predictive likelihood between [0, 1]), and 2) we submit subsets of the triplet [e.g., (*h, r*), (*h, t*), or (*r, t*)] and query the system to identify a linking *h*, *r*, or *t* [i.e., *t* given (*h, r*), *r* given (*h, t*), or *h* given (*r, t*)]. The first is a common approach used to perform LBD using KGC. However, it requires the user to derive all (*h, r, t*) queries to test hypotheses. The second approach generates a list of all known *h*, *r*, or *t* concepts ranked by their related confidence. This approach occurs at the expense of less discriminative information provided as model input. However, its advantages over the first solution include requiring less domain-explicit knowledge from the researcher and fewer resources being exhausted for generating system queries (hypotheses). Existing works such as the *CD-2* method proposed by Crichton et al. (2020) use the first approach while the *MLP* method proposed by Cuffy and McInnes (2023) uses the second. Given both approaches for creating the graph, undirected graphs are used for co-occurrence-based systems, and directed or undirected are used for relational database graphs. Works such as Pyysalo et al. (2018), Crichton et al. (2020), Ding and Jin (2021), Škrlj et al. (2021), and Cuffy and McInnes (2023) explore undirected graphs and Luo et al. (2018), Preiss and Stevenson (2018), Sang et al. (2018a), Sang et al. (2018b), Sang et al. (2019), Zhang et al. (2021), and Daowd et al. (2022) explore directed graphs for LBD.

#### 2.2.2 Embedding nodes in latent space

Incorporating graph theory techniques provides tools to represent complex knowledge using simplistic structures and intuitively visualize and analyze data (e.g., path, connectivity, and centrality analysis). However, embedding the spatial relationships within these graphs into continuous (dense) low-dimensional representations for graph-based analytical tasks remains challenging (e.g., node and link prediction). Unlike Euclidean data (e.g., text and images), graphed data compose structures in non-linear Euclidean space. Since dimensionality reduction relies on preserving spatial properties between graphed elements (nodes) such that connected nodes are near each other in latent space, it makes preserving graph properties in this space difficult. Furthermore, graph constraints can impact the efficacy of the encoding algorithm (i.e., data sparsity, directed vs. undirected graphs, homogeneous vs. heterogeneous graphs, and weighted vs. binary graphs).

There are three primary methods to address these constraints: (1) matrix factorization-based methods, (2) random walk-based methods, and 3) neural network-based (encoder-based) methods. Matrix-factorization methods such as Roweis and Saul (2000), Nickel et al. (2011), and Cao et al. (2015) use one of many matrices (i.e., weighted or binary adjacency, Laplacian, and node transition probability matrix) along with a proximity measure and dimensionality reduction technique to construct node embeddings. However, these methods suffer in capturing higher-order proximity-based relationships between nodes. Furthermore, their computational complexity increases exponentially along with the size of the factored graph. This makes their applicability intractable for large-scale graphs. Random walk-based methods such as DeepWalk (Perozzi et al., 2014) and Node2vec (Grover and Leskovec, 2016) use a different approach to embed graphs into low-dimensional representations. DeepWalk represents a sequence of nodes (i.e., a random walk) where subsequent sequence nodes are selected based on a probabilistic distribution and encodes spatial relationships (structural characteristics) by leveraging the transition probabilities of reaching a given node within the random walk such that nodes co-occurring within the sequence are near each other in latent space (Perozzi et al., 2014). Node2vec (Grover and Leskovec, 2016) improves DeepWalk's unbiased random walk algorithm by introducing parameters that perform biased walks through manipulating the probability of returning to a previous node and performing breath-first search (BFS) or depth-first search (DFS) walks. Both approaches attempt to embed graph structure using sequences of collocation features and later feed their generated sequences into task-specific (downstream) neural networks to learn node representations. Neural network-based methods such as Large-scale Information Network Embedding (LINE) (Tang et al., 2015) and Structural Deep Network Embedding (SDNE) (Wang et al., 2016) use first and second-order proximity information between nodes *within a KG* to preserve spatial relationships (structural characteristics). LINE embeds first and second-order graph structure information using similarities between directly connected nodes. First-order proximity leverages semantics between neighboring nodes, and second-order proximity preserves similarity between neighborhoods within the graph to embed the graph structure into low-dimensional representations. These representations can be combined to improve representation quality. We provide further details of the LINE algorithm in Section 4.1 (LINE). In comparison, SDNE also leverages first and second-order node proximities. However, this approach jointly optimizes both proximities through a series of non-linear layers within a semi-supervised DL model (i.e., an autoencoder) to derive low-dimensional node representations. We use the LINE embeddings in our method in order to perform a direct comparison against previous works.

#### 2.2.3 Evaluating LBD

To evaluate the efficacy of LBD systems for discovering novel knowledge, three common methods are used: (1) domain-expert vetting, (2) discovery replication, and 3) time-slicing. The first method requires domain experts to vet relationships identified by the system. Experts with such skills may be difficult to obtain, time management or project deadlines may present further obstacles, and high resource requirements may negate this method. In comparison, discovery replication includes replicating the conditions to re-discover (or reproduce) one or many known relationships. This is the most common and cost-effective method to evaluate new LBD systems. In this method, we focus on replicating a single relationship or a set of relationships. For either task, we LINE embeds first and second-order graph structure information using similarities between directly connected nodes. First-order proximity leverages semantics between neighboring nodes, and second-order proximity preserves similarity between neighborhoods within the graph to embed the graph structure into low-dimensional representations. These representations can be combined to improve representation quality. time-slice our data by selecting a cutoff year and split our graph data into two sub-graphs: pre-cutoff (training set) and post-cutoff (evaluation set). When replicating a single relationship, our cutoff year represents the known year of discovery. We train our systems using the pre-cutoff data and evaluate our system using the post-cutoff data. For single relationship discovery replication (SRDR), we query our system to identify the known relationship as implicit with high predictive likelihood and rank the true relationship among a set of relationships. When replicating a set of relationships (e.g., multi-relationship discovery replication), we evaluate the system's ability to replicate all known relationships and use one or more of the following metrics to determine system performance: Precision, Recall, F1-Score, Precision@K, Mean Average Precision@K, and Mean Reciprocal Rank (MRR). Time-slicing follows a similar methodology, however we evaluate the system's ability to reproduce all known relationships within the evaluation set. In this work, we evaluate the performance of our method using SRDR and compare our work to existing methods using mean rank.

## 3 Data

We train and evaluate our method using the Hallmarks of Cancer (HOC) datasets (Pyysalo et al., 2018). These datasets focus on five recent discoveries involving the molecular biology of cancer and support both closed and open LBD. We refer to these datasets as HOC1, HOC2, HOC3, HOC4, and HOC5. Table 1 shows statistics for each dataset. All five datasets have been extracted from PubMed (1996) articles between the years of 2006 and 2016 using PubTator (Wei et al., 2013). They contain explicit *A-B-C* relationships expressed as grounded concept triplets. These concepts are extracted from a variety of sources such as diseases and chemicals from the Chemical Entities of Biological Interest (ChEBI) (Degtyarenko et al., 2007) and Medical Subject Heading (MeSH) (Lipscomb, 2000) databases, genes and proteins from the NCBI Gene database (Maglott et al., 2005), and names of species from the NCBI Taxonomy (Federhen, 2012). Shown in Table 2, all datasets focus on identifying a known relationship (i.e., discovery replication), and are time-sliced into training and evaluation sets using the known year of discovery. Each concept triplet is ranked using the Jaccard similarity coefficient. This score expresses the degree of similarity between the *A* → *B* and *B* → *C* paths within the complete *A* → *B* → *C* relationship. They are used to indicate positive and negative samples within each dataset. *A* → *B* → *C* relations with a Jaccard similarity coefficient of zero signify negative samples while scores greater than zero indicate positive samples. All training sets within each dataset maintain a 50%-50% distribution of positive-to-negative samples while the evaluation set contains only positive samples.

**Table 1:** Dataset statistics.

**Name**	**HOC1**	**HOC2**	**HOC3**	**HOC4**	**HOC5**
**Training statistics**					
Number of positive samples	100,000	100,000	100,000	100,000	100,000
Number of negative samples	100,000	100,000	100,000	100,000	100,000
Total number of samples	200,000	200,000	200,000	200,000	200,000
Number of unique A concepts	114,189	114,323	111,389	100,490	116,887
Number of unique B concepts	90,700	90,483	88,753	81,165	92,412
Number of unique C concepts	94,517	94,440	92,640	84,998	96,427
Number of unique concepts	151,257	151,093	145,098	123,002	157,239
Number of unique A-B relations	194,655	194,726	194,477	193,410	194,846
Number of unique B-C relations	199,931	199,924	199,935	199,946	199,925
Number of multi-B concept links	409	352	378	476	371
Max multi-B concept link size	3	3	2	3	3
**Evaluation statistics**					
Number of Samples	2,294	654	425	444	1,049
Number of unique A concepts	1	1	1	1	1
Number of unique B concepts	2,294	654	425	444	1,049
Number of unique C concepts	1	1	1	1	1
Number of unique A-B relations	2,294	654	425	444	1,049
Number of unique B-C relations	2,294	654	425	444	1,049

Number of Positive Samples: Describes the number of samples which have the Jaccard similarity coefficient greater than 0. Number of Negative Samples: Describes the number of samples which have the Jaccard similarity coefficient of 0. Number of Multi-B Concept Links: Describes the number of *A-to-C* links which are associated with more than one *B* concept. Max Multi-B Concept Link Size: Describes the maximum number of linking *B* concepts associated with a given *A-B-C* link in the dataset.

**Table 2:** True *A-B-C* closed discovery reduplication links by dataset.

**Name**	**A Concept**	**B Concept**	**C Concept**
HOC1^a^	NF-κB (PR:000001754)	Bcl-2 (PR:000002307)	Adenoma (MESH:D000236)
HOC2^b^	NOTCH1 (PR:000011331)	senescence (HOC:42)	C/EBPβ (PR:000005308)
HOC3^c^	IL-17 (PR:000001138)	p38α (PR:000003107)	MKP-1 (PR:000006736)
HOC4^d^	Nrf2 (PR:000011170)	ROS (CHEBI:26523)	pancreatic cancer (MESH:D010190)
HOC5^e^	CXCL12 (PR:000006066)	senescence (HOC:42)	thyroid cancer (MESH:D013964)

^a^Bcl-2 is a critical mediator of intestinal transformation (Van Der Heijden et al., 2016).

^b^Notch1 mediates a switch between two distinct secretomes during senescence (Hoare et al., 2016).

^c^Integrating p38αmapk immune signals in nonimmune cells (Gaffen and McGeachy, 2015).

^d^Oncogene-induced nrf2 transcription promotes ros detoxification and ^e^tumorigenesis (DeNicola et al., 2011).

^e^Senescent tumor cells lead the collective invasion in thyroid cancer (Kim et al., 2017).

## 4 Materials and methods

In this section, we describe our method. First, we present our DL model and discuss its architecture. Second, we discuss how we represent terms as concepts and concepts as embedded representations fed into our model. Third, we discuss how we represent our model's output as concept embeddings to replicate five known HOC discoveries. Finally, we discuss how we use discovery replication to evaluate the efficacy of our method.

### 4.1 LINE

Large-scale Information Network Embedding (LINE) (Tang et al., 2015) is a neural network-based embedding method that learns to embed graph vertices into low-dimensional representations. While previous graph embedding works rely on the traditional implementation of stochastic gradient descent (SGD) as the model's optimization objective, LINE uses an edge-sampling optimization approach to improve the model's learning objective, inference effectiveness, model efficiency, and learn vertex embeddings within undirected, directed, and weighted graphs. Given an arbitrary graph defined as *G* = (*V, E*) where V is the set of vertices and E is the set of edges between vertices, edges *e* ∈ *E* form ordered weighted pairs that indicate the strength of the connection (i.e., a binary or any non-negative value). For each *v*_*i*_ ∈ *V*, LINE learns to encode local pairwise proximities between *v*_*i*_ and its immediate neighboring vertices *v*_*j*_ (connected by an edge) in low-dimensional space (i.e., first-order proximity). It accomplishes this using a conditional joint probability and an empirical probability between *v*_*i*_ and all *v*_*j*_ neighbors, and KL Divergence to minimize the pairwise distance between the two distributions such that pairwise similarities are greater between neighboring vertices *v*_*i*_ and *v*_*j*_. However, this method only supports undirected graphs and cannot preserve complex graph structures. To address this limitation, LINE also explores second-order proximities for all *v* ∈ *V* to induce the embedding of the graph structure in its representations. This approach preserves similarities between vertices up to two hops away (i.e., preserves neighborhood information between vertices using a friends-of-friends network). LINE is scalable to graphs containing millions of nodes, and successfully learns to embed first and second-order proximity information (graphs structure) within its vertex embeddings. Tang et al. (2015) found the most effective approach for generating vertex embeddings entails generating both sets of embeddings (first and second-order), concatenating them into a longer set of low-dimensional embeddings, and balancing them by re-weighting. We utilize this approach with the LINE algorithm as a basis for our work.

### 4.2 Base model

**Figure 2:**
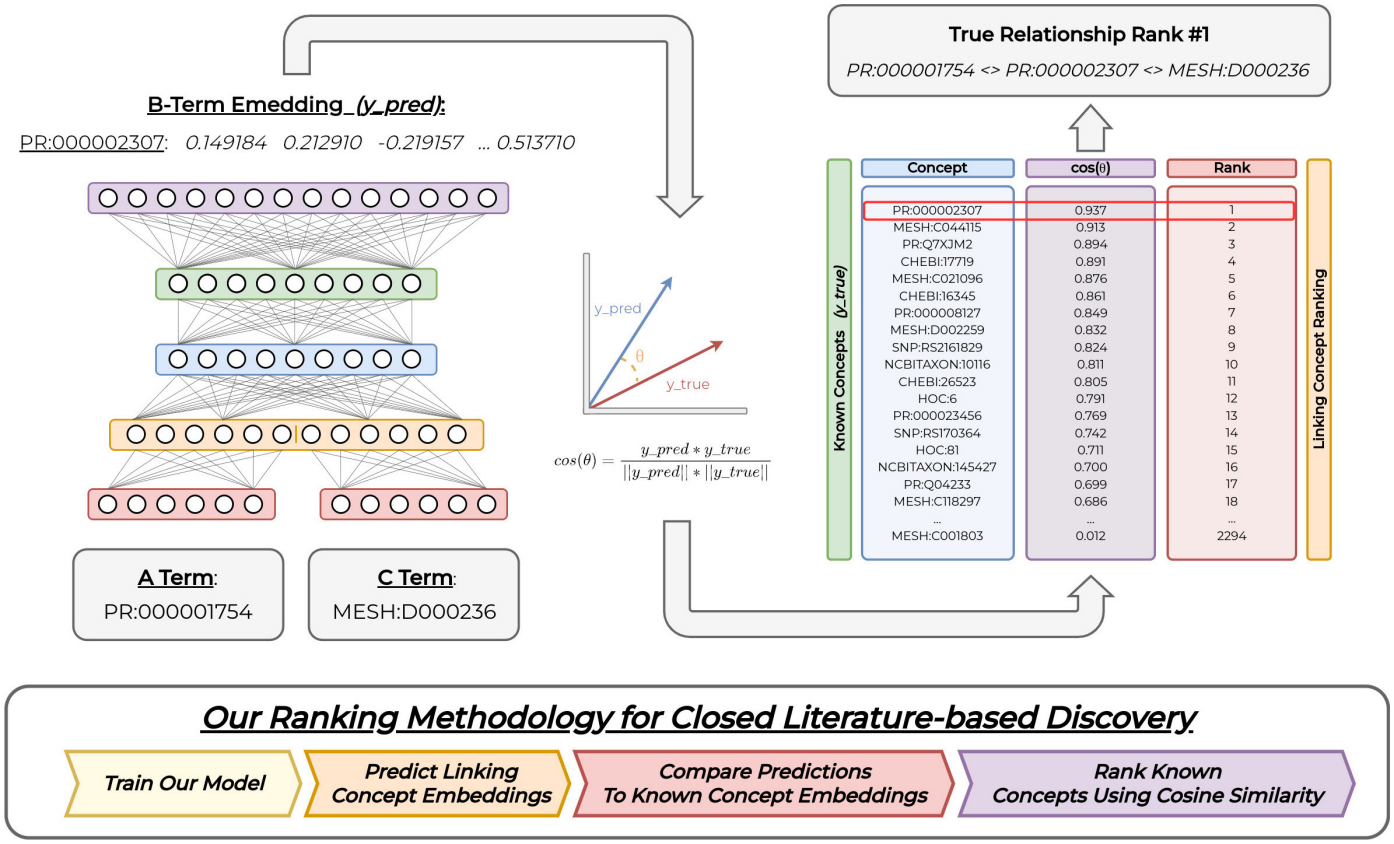
Overall model. We provide *A* and *C* concept embedding representations as input into our model. The model outputs a related linking *B* concept embedding representation. We compare this predicted concept representation to all known *B* concepts using cosine similarity, and perform closed discovery concept ranking using these cosine values. Our model figure demonstrates concatenated input representations. However, we explore three types of input representations. Our figure shows a model trained on the HOC1 dataset, both input concepts, the predicted linking concept representation, and all computed concept similarity scores used to replicate the HOC1 relationship. Full acyclical connections between subsequent layers are not shown for ease of visualization.

Figure 2 shows our four-layer multi-layer perceptron (MLP) base model architecture. Our model's input layer accepts embeddings to represent *A* and *C* concepts and transforms inputs using our desired scaling factor with four approaches to represent edges between inputs (i.e., averaging, Hadamard, or concatenation). Our output layer produces embedded representations for our *B* concepts that matches the embedding dimensions of our input concepts. We provide further details of our model architecture in Section 5 *(Experimental Details)*.

The HOC datasets contain co-occurring gene, disease, protein, and chemical terms extracted from PubTator (Wei et al., 2013) into time-sliced knowledge sub-graphs. We represent terms using grounded concepts as vertices and use co-occurrence between concepts as the edges. To train our model, we represent graph co-occurrence information as *A-B-C* relations. We embed semantics between immediate and neighboring concepts in the knowledge graphs constructed from each dataset using the *Large-scale Information Network Embedding* (LINE) (Tang et al., 2015) algorithm. Our model receives *A* and *C* concept representations as input and produces a single *B* concept embedding representation as output. Given our model input, we train our model to complete the closed discovery path by predicting an embedding that links the two input concepts. To achieve this, our model exploits relatedness between embeddings to extract relevant and discriminative features used to identify explicit (and implicit) relations between the input (*A* & *C*) concepts and our desired linking *(B)* concept. We train our model to generalize this embedded semantic information between our input and output concepts using cosine similarity loss vs. traditional crossentropy loss. Thus, our model learns to use similarity between concepts to yield related linking *B* concepts for implicit relation discovery.

### 4.3 Input representation

We represent *A* and *C* concepts as static embeddings generated from the LINE (Tang et al., 2015) algorithm as input into our model. To represent co-occurring links between concepts in the graph, we use three common approaches: (1) computing the *average* among embeddings, (2) *concatenating* both embeddings, and computing the *Hadamard* product among embeddings. *Averaging* computes the mean embedding among all inputs, *concatenation* combines all input representations into a larger representation, and *Hadamard* computes the element-wise multiplication among all dimensions between inputs. Each approach results in a single representation that is fed into our model. Previous work has shown multiplying the input features by a factor of 10 induces inductive biases during model training to improve generalization (Crichton et al., 2020). Therefore, we also explore multiplying our input embedding representations by a factor of 10.

### 4.4 Output representation

Standard DL objectives include training models to classify input(s) into discrete classification labels (e.g., binary and multi-class classification). However, our training objective differs from this approach. We use a similarity-based objective function to train our model to predict an embedding given a set of inputs. For a given *A* and *C* input representation, our model predicts a *B* concept embedding that links the input concepts for testing hypotheses. To achieve this, we use the hyperbolic tangent activation function to bound the range of our model's output logits between [−1, 1].

### 4.5 Evaluation

We show our evaluation methodology in Figure 2. All HOC datasets have been time-sliced for the task of discovery replication. We evaluate our method by simulating the conditions necessary to re-discover each known (explicit) *A-B-C* relationship implicitly. First, we train our model over a given HOC training dataset to predict each *B* concept (vertex) embedding that links a set of inputs (*A* & *C*). Given our model's learning objective, we then evaluate its performance using a ranking approach. In this approach, we provide the *A* and *C* from the true relationship as input into our model. Next, we use cosine similarity to compare the model's predicted *(B)* concept embedding against the embedding representations for all known (unique) *B* concepts within the evaluation set. We rank these computed cosine similarity values for each concept in descending order such that the most similar (or related) concept occurs first within the list of sorted elements (i.e., list of *B* concepts). This implies that the most similar *B* concept embeddings to the model's predicted output will be ranked numerically lower when compared to their cosine similarity values (i.e., an inverse relationship). To determine evaluation performance, we report the rank (index) of the gold *B* concept within the sorted list. We consider the SRDR task as successful if the gold *B* concept is ranked numerically lower among all remaining concepts (i.e., ideally an index value near 1 for SRDR). However, the system may identify other implicit but relevant relationships in addition to our desired gold *B*. We show an example of this ranking approach on the right-side of Figure 2. We repeat this process for all pairs of true SRDR relationships and matching datasets.

## 5 Experimental details

Our data preprocessing steps include lowercasing all text prior to generating concept embeddings. We generate our embeddings using the Large-scale Information Network Embedding (LINE) (Tang et al., 2015) algorithm. This algorithm offers four main hyperparameters to generate embeddings: order, sample, size, and number of threads. To represent each concept in the vocabulary for each dataset, we generate two sets of embeddings: first and second-order embeddings. We provide a description of the LINE algorithm in Section 2.2.2 *(Embedding Nodes In Latent Space)*. We use the following settings to generate the concept embeddings: 10 threads, size of 50 and 1,000 samples. For first-order, we set the *order* parameter to 1, and for second-order we set the *order* parameter to 2. We then concatenate both sets of embeddings to form our unique set of 100-dimensional concept embeddings for each HOC dataset.

We build our LBD experimental framework, Neural Network Architectures for Literature-Based Discovery (NNLBD),^1^ using Tensorflow version 2.9 (Abadi et al., 2015) and Python 3.10.11. For our MLP model, we utilize the number of embedding dimensions from our input concepts as number of hidden units at each layer. We train our models on NVIDIA Tesla V100 PCIe 32 GB GPUs using the standard ADAM optimizer with cosine similarity loss and the hyperbolic tangent activation function at the output layer. Our training settings include a learning rate of 1e-4. We use a batch size of 256 and dropout is set to 10%. For the number of training epochs, we found some combinations of datasets and input representations reached optimal convergence on the evaluation set after 5 epochs while others needed 400 epochs. We found the stability of our earlier converging models remained consistent with increased training iterations. Therefore, we train all of our models for 400 epochs. We evaluate our models after every training epoch and report the ranking of the *B* concept within the true *A-B-C* link among all *B* concepts in the evaluation set vocabulary. For our input scaling experiments, we multiply our *A* and *C* concept representations by a factor of 10 before forward propagating through the neural network. Our non-input scaled experiments do not perform this modification.

## 6 Results

In this section, we show and discuss our results, and compare our method to previous works using two types of vocabularies: (1) *evaluation* and (2) *comprehensive* vocabularies. The *evaluation* vocabulary contains all unique *B* concepts within each evaluation dataset (i.e., list of filtered *B* concepts by semantic type), and the *comprehensive* vocabulary contains all known (unique) *B* concepts (i.e., complete list of known *B* concepts). We evaluate over two vocabularies to provide an in-depth and direct comparison against previous work (i.e., the *CD-2* method only reports performance over the *evaluation* vocabulary). We identify statistically significant performances by grouping significant performances among all compared methods using their *evaluation* vocabularies. Lastly, we provide an analysis of the architectural differences between each compared model and discuss the merits of each method.

We also explore scaling our input representations to improve inductive biases during model training and expedite convergence. Previous works such as Crichton et al. (2020) and Cuffy and McInnes (2023) have shown performance improvements when performing this approach. However, we found no significant difference between scaling our input representations by a factor of 10 and not performing this approach for our task. To compare and detect at least one pair of significant performances between both approaches, we use the method described by Demšar (2006). We use the non-parametric Friedman Test (NPFT) (Friedman, 1940) to determine if a significant performance exists among at least one pair of models using their *evaluation* vocabularies and the Nemenyi *Post-Hoc* Test (NPHT) (Nemenyi, 1963) to group models by significant performances. For the NPFT, we use the following parameters: *k* = 6 models (3 input scaled and 3 non-input scaled representations) and *N* = 5 datasets. We obtain our critical value (CV) by computing our degrees of freedom(s) as (*k*−1) and (*k*−1)(*N*−1) which provides the *CV* = 2.71 using the F-Distribution table with a significance level of α = 0.05. We utilize the following hypotheses to determine significant performances:

- *H*_0_: There are no significant performances among all models.
- *H*_*A*_: There is a significant difference between at least one pair of models.

We compute our Friedman statistic (*F*_*F*_) as 0.5573. Since our *F*_*F*_ < *CV* we reject our *H*_*A*_ hypothesis and accept the *H*_0_ hypothesis. Thus, there is no significant difference between the two approaches to represent input. We report the scaled input results as a basis for further analysis and comparison against previous works.

### 6.1 Comparison with previous work

Table 3, shows the performance of our model over the Hallmarks of Cancer datasets, compared to the best performing methods described by Cuffy and McInnes (2023) [MLP (CD)] and Crichton et al. (2020) (CD-2) (i.e., *feature-scaled* input representations). The *CD-2* model is a shallow two-layer neural network where the input is a set of vertex triplets (i.e., *A-B-C*) within the KG. The first (hidden) layer contains 100 units, and the second is an output layer that uses the softplus (Nair and Hinton, 2010) activation function and binary crossentropy to output a score indicating the likelihood that edges exist between all inputs (i.e., strength of the connection). Compared to the CD-2 model, *MLP (CD)* uses a five-layer DL (MLP) model to identify implicit relationships where the model input is a pair of KG vertices (*A* & *C*), and each hidden layer contains at least 200 units. Similar to the CD-2 model, binary crossentropy is used to generalize the model's training objective, and sigmoid activation is used to restrict the upper and lower bounds between [0, 1]. This model predicts scores among all *B* concepts in the vocabulary which indicates the likelihood that edges exist between the input concepts (*A* & *C*) and each known *B* concept. Our method combines the advantages of both prior approaches for SRDR and uses cosine similarity to generalize the training objective. We provide more details of our base model architecture in Section 4.2 *(Base Model)*, and details of our input and output representation approaches in Sections 4.3 *(Input representation)* and 4.4 *(Output representation)*.

**Table 3:** Our model vs. previous works.

		**Rank**				
		**Evaluation vocabulary**			**Comprehensive vocabulary**	
**Dataset**	**Input type**	**OM**	**MLP (CD)**	**CD-2**	**OM**	**MLP (CD)**
HOC1	Average	**4.2**	18.0	50.4	**4.6**	19.0
	Concat	**5.8**	14.6	26.2	**6.2**	17.6
	Hadamard	4.4	25.2	**1.4**	**4.4**	27.2
	Mean	**4.8**/2,294	19.3/2,294	26.0/2,294	**5.07**/29,903	21.27/29,903
HOC2	Average	101	205.2	**5.8**	**147**	350
	Concat	106.4	170.4	**7.4**	**146.6**	275.8
	Hadamard	121.8	210.6	**13.4**	**185.6**	384.4
	Mean	109.7/654	195.4/654	**8.9**/654	**159.73**/29,969	336.73/29,969
HOC3	Average	24.8	**14.4**	76.6	**34**	35.4
	Concat	56.6	**16.4**	123.2	99.8	**37.2**
	Hadamard	28.2	**23.0**	254.2	**35.8**	53.2
	Mean	36.5/425	**17.9**/425	151.3/425	56.53/29,253	**41.93**/29,253
HOC4	Average	**112.6**	300.0	256.2	**289.4**	300
	Concat	**68**	234.4	115.8	**132.6**	234.4
	Hadamard	117.4	323.4	**21.0**	**301**	323.4
	Mean	**99.3**/444	285.9/444	131.0/444	**241**/26,393	285.93/26,393
HOC5	Average	254.4	179.6	**67.2**	410.8	**239.2**
	Concat	205.2	166.8	**84.4**	329.8	**209.8**
	Hadamard	171.4	**247.8**	597.4	**309.2**	417.2
	Mean	210.3/1,049	**198.1**/1,049	249.7/1,049	349.93/29,903	**288.73**/29,903

We compare mean ranks among five runs for our *averaged, concatenated*, and *Hadamard* input representations for our model (OM), to the methods described by Cuffy and McInnes (2023) [MLP (CD)] and Crichton et al. (2020) (CD-2). We report the best performances for all compared methods (i.e., *feature-scaled* ranked performances for the *CD-2* and *MLP (CD)* methods). We separate performances using two discrete vocabularies: (1) *evaluation* and (2) *comprehensive*. The *evaluation* vocabulary contains all unique *B* concepts within an evaluation dataset (i.e., filtered concepts by semantic type). The *comprehensive* vocabulary contains all known (unique) *B* concepts within the entire vocabulary (i.e., complete listing without filtering). We indicate the best performing models, separated by vocabulary type, in bold.

When comparing the *evaluation* vocabulary performances of each method, we found the results varied depending on the method of comparison chosen. Comparing the averaged performances among all input representations shows our method performs optimally on the HOC1 and HOC4 datasets using the averaged and concatenated representations. The *CD-2* model achieves the best performance on the HOC2 datasets for all input representations, and the best performance on the HOC5 dataset using the averaged and concatenated representations. The *MLP (CD)* model outperforms the remaining models on the HOC3 dataset for all representation approaches and across the HOC5 dataset using Hadamard inputs. When comparing mean performances among all input representation approaches for all three methods, our results show our method performs the best on the HOC1 and HOC4 datasets. The *MLP (CD)* model performs the best on the HOC3 and HOC5 datasets while the *CD-2* model performs the best on the HOC2 dataset. We found the latter of these results differs from the former due to outlier performances when computing the mean among all input representations for each method. Thus, careful consideration and precautions must be exercised when selecting the appropriate input representation approach for the *CD-2* method. We found variance between averaged ranks is high as seen within the HOC5 performance metrics. In comparison, our method achieves lower variance between input representation approaches when comparing the performances over the same dataset.

To determine and group significant performances among all compared methods using their *evaluation* vocabularies, we use the NPFT with our aforementioned hypotheses. However, for this test, we're comparing all input representation approaches among all three methods. This results in nine total models. Thus, our *k* = 9 and *N* = 5 for the NPFT. We determine our critical value (CV) to be 2.27 for (*k*−1) and (*k*−1)(*N*−1) degrees of freedom using the F-Distribution table with a significance level of α = 0.05. We compute the Friedman Statistic (*F*_*F*_) as 0.4183, and found we must reject the *H*_*A*_ hypothesis and accept the *H*_0_ since *F*_*F*_ < *CV*. Therefore, the NPFT shows no significant groupings among compared input representations over all compared methods. This indicates all methods perform similarly.

To demonstrate the effectiveness of our method, the right side of Table 3 shows our model performances when comparing against all known *B* concepts when identifying each true HOC relationship triplet (i.e., the *comprehensive* vocabulary). Despite the large increase in the number of compared concepts (e.g., 29,903 *comprehensive* vs. 2,294 *evaluation* vocabulary concepts for HOC1), we found our method outperforms the *MLP (CD)* method over the HOC1, HOC2, and HOC4 datasets. For the HOC3 dataset, our method performs best using the *average* and *Hadamard* input representation approaches. Lastly, over the HOC5 dataset, our method outperforms the *MLP (CD)* method using the *Hadamard* approach.

**Figure 3:**
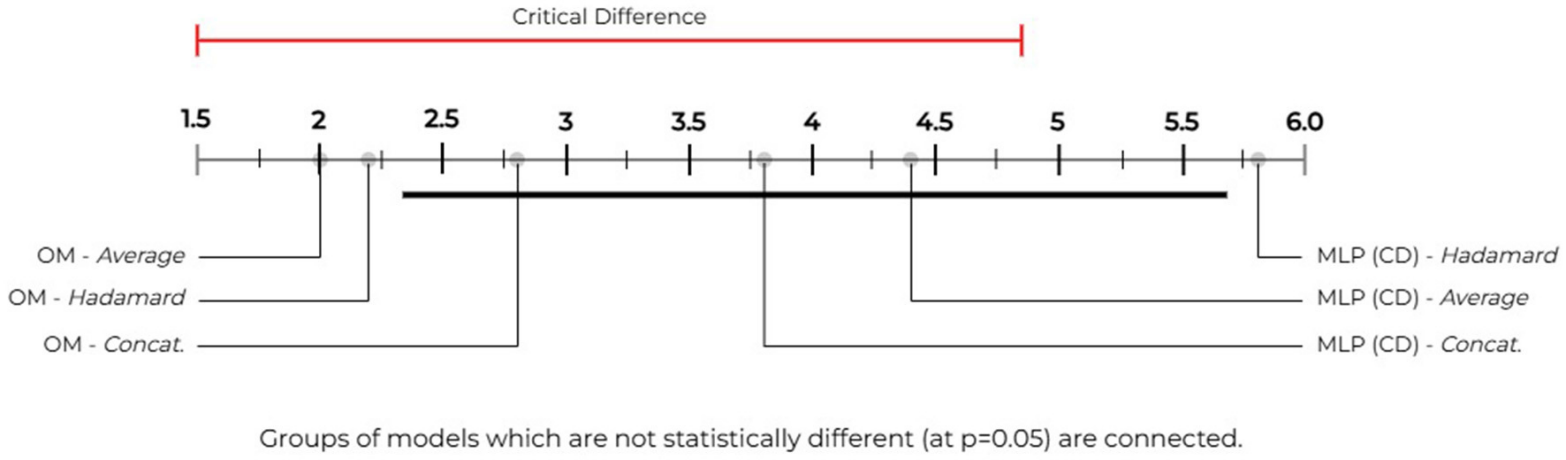
Our model vs. MLP (CD)—statistical significance groupings: we use the Nemenyi *post-hoc* test (Nemenyi, 1963) to compare our method against the *MLP (CD)* method described by Cuffy and McInnes (2023) and group statistically significant performances over the *comprehensive* vocabularies. We determine our critical difference to be 3.3717 (i.e., the length of the red and black bars). Pairwise differences between performances that exceed this bar (or value) are considered statistically significant. We use the *black bar* to group performances that are not statistically different. Our figure shows that our method's *averaged* and *Hadamard* approaches outperform the *MLP (CD)* method's *Hadamard* approach by a statistically significant margin.

To perform significance testing among the *comprehensive* vocabulary models, we use the following parameters with the NPFT and our aforementioned hypotheses (*H*_0_ & *H*_*A*_): *k* = 6 models [3 *OM* and 3 *MLP (CD)*] and *N* = 5 datasets. We obtain our critical value (CV) by computing our degrees of freedom(s) as (*k*−1) and (*k*−1)(*N*−1) which provides the *CV* = 2.71 using the F-Distribution table with a significance level of α = 0.05. We compute our Friedman statistic (*F*_*F*_) as 6.1744. As our *F*_*F*_>*CV* we accept our *H*_*A*_ hypothesis and reject the *H*_0_ hypothesis. Therefore, there are at least one pair of pairwise significant performances among these approaches over the *comprehensive* vocabularies. Figure 3 shows the model groupings by statistically significant performances. We use the NPHT with our *k* = 6, the *Studentized Range q Table*, and determine our critical value to be 2.850 (i.e., $(k,\inf)/\sqrt{2}\to 4.03/\sqrt{2}$) and the critical difference as 3.3717 for α = 0.05. The NPHT shows that our method's *averaged and Hadamard* approaches significantly outperforms *MLP (CD)'s Hadamard* approach with no other significant differences noted among the remaining models.

### 6.2 Discussion

**Figure 4:**
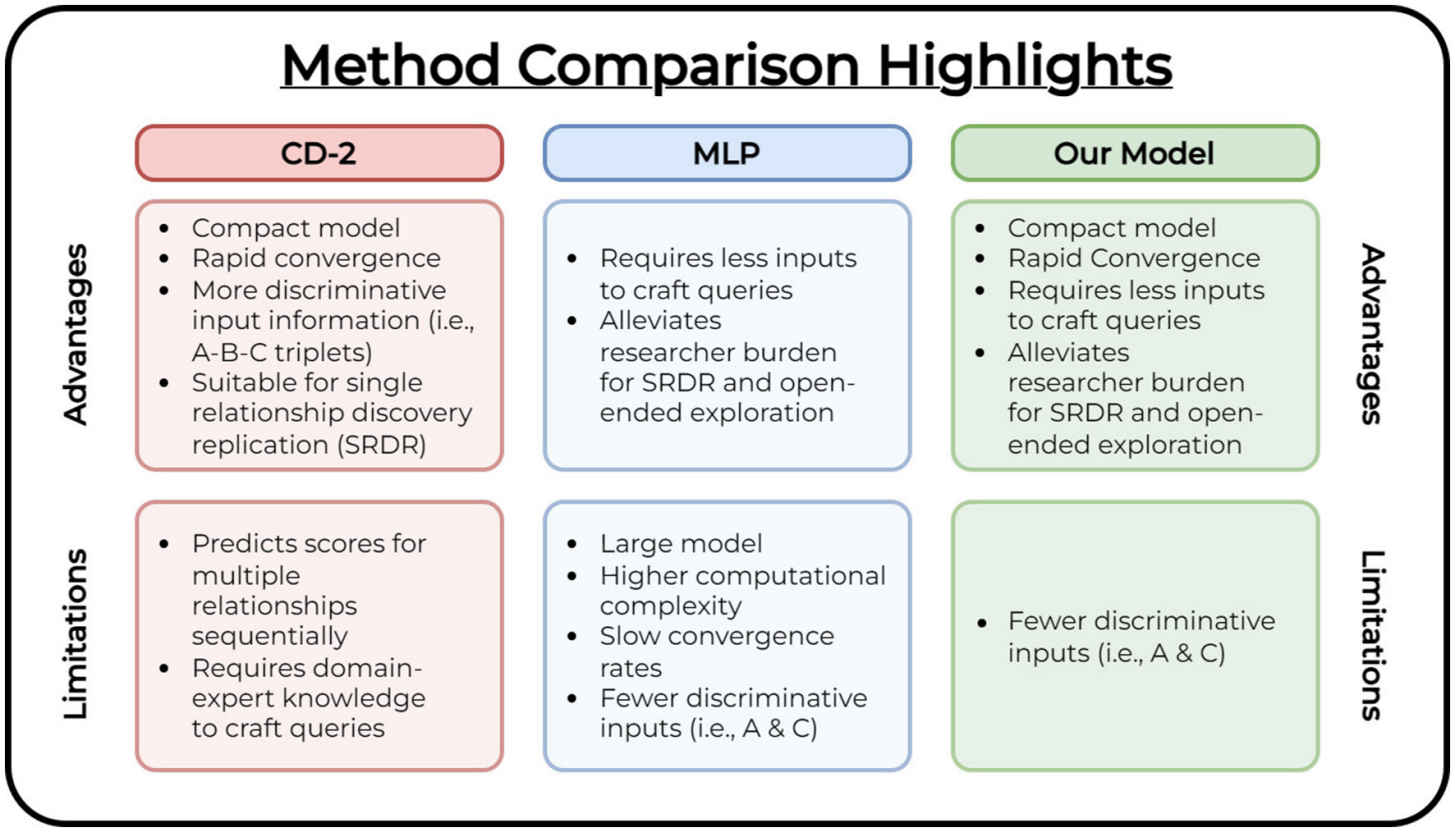
Method comparison table. We highlight the overall advantages and limitations of each compared method.

Figure 4 lists the overall advantages and limitations among all compared methods. While no significant difference exists between all compared methods for SRDR assessment of the *evaluation* vocabularies, it is imperative to highlight the merits of our method's technical contributions and advantages for open-ended research.

The *CD-2* (Crichton et al., 2020) method utilizes a compact model to perform the task of triplet link prediction among concepts (i.e., predict edges among a set of concept triplets) and performs well for SRDR. However, it requires users to construct expert-crafted queries to facilitate open-ended exploration. Construction of these queries is a time-consuming process that requires explicit domain knowledge. The *MLP (CD)* (Cuffy and McInnes, 2023) method alleviates this prerequisite by reducing the high domain knowledge requirement for constructing queries and produces a listing of fruitful relationships to the user as a ranked list (i.e., it only requires the user to provide a starting and target concept as input, and produces a set of potential linking terms along with their confidence scores). For this model to achieve similar performance to *CD-2*, it requires a significant increase in the number of model parameters. This leads to greater computational complexity which indicates more training is necessary to converge as we introduce new data to the model. Our method combines the advantages of both compared works. We explore a compact model (i.e., a reduced number of parameters compared to the *MLP (CD)* model) that converges in as low as 5 epochs compared to the MLP's 800. Compared to the *CD-2* model, our linking concept representation prediction approach requires fewer discriminative inputs to achieve similar performance and provides an avenue to leverage the primary advantage demonstrated in the *MLP (CD)* method. It requires less domain-explicit knowledge to explore and derive relationships of interest by providing a ranked list of linking concepts while achieving comparable performance to both compared studies. This is shown in our assessment of both types of vocabularies (i.e., *evaluation* and *comprehensive*).

To analyze the efficacy of our method for SRDR of the five known HOC relationships, we compare the true performances of our method to ten randomly generated relationships for each dataset. We list these random relationships in Supplementary material document. Our rationale behind this evaluation method is to show the difference in ranking performance between both types of relationships (i.e., true vs. random). Since higher ranking values equate to lower similarity scores (i.e., an inverse relationship), it suggests that our model predicts the likelihood of edges existing between the random triplet's vertices is low. We theorize the random relationships will result in higher numerical rankings when compared to our known relationships (i.e., higher ranking values). To accomplish this, we follow the procedure and use the *A-B-C* random relationships as defined in Cuffy and McInnes (2023). We ensure that all generated *A-B-C* link concepts are unique and no repeat relationship triples are evaluated. We scale our inputs by a factor of 10 using the *concatenated* approach for representing input, and report the mean rank among all ten random relationships for each dataset. All remaining hyperparameters follow our previous training and evaluation methods to determine system performance.

When compared to the true relationships, we found the random relationships produced higher ranking values with a minimal number of ties. For HOC1, this results in a mean rank of 14549.6. HOC2 performs the best among all datasets achieving a mean rank of 9958.9. HOC3 and HOC5 performs similar to HOC1 achieving ranks of 12611.7 and 13507.3 respectively. Lastly, HOC4 performs the worst with a mean rank of 15299. These results support our initial remarks and validate our findings for our discovery replication methodology to demonstrate the efficacy of our method.

## 7 Conclusion, limitations and future work

In the section, we discuss our conclusions and provide several areas for future work.

### 7.1 Conclusion

In this work, we introduce and explore a novel approach for knowledge graph completion (KGC) in the context of closed literature-based discovery (LBD), leveraging Unified Medical Language System (UMLS) concepts for term representation. Unlike traditional KGC models, which often require explicit domain knowledge and rely heavily on predefined triplets, our method shifts the focus to implicit relationship discovery by predicting the embedding of the intermediate concept A-B-C triplet using multiple edge representation techniques: averaging, concatenation, and Hadamard product. This innovative use of concept embedding prediction represents a departure from typical classification tasks and introduces a more efficient pathway to identifying hidden relationships.

A key distinction of our approach lies in the semantic-based learning of relationships, where we aim to capture grouped concept similarities within the graph to facilitate the discovery of implicit links. By reducing the need for expert-generated queries, we lower the barrier to knowledge discovery, enabling researchers to explore new connections with less prior domain expertise. Additionally, our method introduces a significant improvement in the generalization speed of relationship discovery, reducing computational overhead while achieving competitive performance compared to existing models.

We evaluate our approach on the Hallmarks of Cancer (HOC) dataset, which is structured to capture explicit and implicit relationships. Our model successfully replicates known discoveries by identifying plausible A-B-C connections, demonstrating its effectiveness in systematic knowledge discovery replication. Moreover, the ability to rank all known concepts against input pairs further distinguishes our method from existing systems, as it supports open-ended exploration and highlights conceptual bridges that may not be immediately apparent in traditional LBD methods.

Our contributions include:

- Introducing a novel concept embedding prediction approach that simplifies the task of uncovering hidden relationships in knowledge graphs.
- Reducing the reliance on manual domain knowledge in hypothesis generation by automatically ranking plausible linking concepts.
- Achieving comparable performance to existing KGC methods while improving efficiency in generalization and reducing the input complexity for concept discovery.

We discuss these innovations in detail, comparing our method to prior works, and highlight how it advances the state of the art in closed LBD.

### 7.2 Limitations and future work

We found our system predicts concept embeddings that can be used for identifying implicit relationships for closed LBD. However, we have identified several limitations of our method. First, our method is trained using cosine similarity loss. This training objective leverages pretrained static concept embeddings to link related semantic spaces to discover new knowledge. In the current implementation of our method, we do not train against negative samples. We believe our results can be improved and method can scale to larger KGs by backpropagating against intelligently selected negative training samples. This can be performed by verifying the generated negative samples do not exist within the evaluation set. Next, our method does not address out-of-vocabulary terms which contradicts its usefulness for adapting to unknown (or new) concepts. To mitigate this concern, retraining the model on new data will be required, or large language models (LLMs) can replace the embedding generation method (LINE) used within this work. However, careful considerations must be expressed when selecting an LLM. These models are pre-trained on a large number of corpora which may violate the time-slicing constraint for discovery replication (i.e., the LLM may have explicitly seen the implicit relationship your discovery replication method is trying to rediscover in the training corpora). This can lead to confirmation bias due to data leakage during model evaluation. Additionally, resource constraints (financial and hardware) may limit the applicability of these architectures and inhibit researchers from pre-training new LLMs for time slicing tasks. Finally, we utilize the HOC datasets and static embeddings as the basis of our work. These datasets perform an *a-priori* preprocessing step that maps terms to concepts within the UMLS (i.e., concept mapping). While this step is used to reduce the vocabulary size, eliminate spurious terms, and address word homonymy, heteronymy, homographs and polysemy, it produces smaller KGs by reducing the granularity of lexical semantics represented within the literature that is used to train our embeddings. Furthermore, the embedding generation algorithm (LINE) used in our work presents a robust approach to represent large KGs during the time of release (i.e., 2015). With today's larger KGs, LINE may experience scalability issues (i.e., preserving complex structural characteristics with larger KGs) and experience information loss during the dimensionality reduction step while encoding their node representations. Moreover, LINE also experiences issues encoding directed graphs (i.e., its first-order proximity KL-Divergence-based conditional joint distribution and empirical probability distribution optimization approach) and does not encode use-specified edge/node attributes. Reworking our model architecture by incorporating alternative graph (e.g., graph attention or graph convolution networks) or DL-based embedding generation algorithms (e.g., contextual embeddings via pre-trained LLMs) can address these concerns. These revisions, along with our stated limitation mitigation approaches, can be adapted to address the reduction in lexical granularity when mapping terms to UMLS concepts and promote representations that embed higher quality lexical semantics to potentially improve evaluation performance.

## Data Availability

The Hallmarks of Cancer datasets explored in this work were curated, maintained, and hosted online by the original authors (Pyysalo et al., 2018).

## Footnotes

^1^ https://github.com/NLPatVCU/NNLBD
