# Supplementary material for: Predicting implicit concept embeddings for singular relationship discovery replication of closed literature-based discovery
Source: Front Res Metr Anal. 2025 Mar 5;10:1509502. doi: 10.3389/frma.2025.1509502 (PMC11920161; doi:10.3389/frma.2025.1509502)
Supplement: Supplementary file 1 [file Data_Sheet_1.pdf]

Supplementary Material

1 RANDOM A-B-C RELATIONS

We evaluate the efficacy of our method by comparing the true *A*-*B*-*C* relation evaluation rankings, among all HOC datasets, to a set of randomly generated *A*-*B*-*C* relations. We list these randomly generated A-B-C relations in Tables S1-S5

Table S1. *Random Experiments*. HOC 1

Dataset	A Concept	B Concept	C Concept
HOC1	CHEBI:23806	CHEBI:38597	PR:000014047
	NCBITAXON:104760	PR:000009321	MESH:D009281
	CHEBI:64683	MESH:C018674	PR:000009268
	MESH:C449580	SNP:RS987525	SNP:RS2494746
	SNP:RS61443	CHEBI:32030	MESH:D002015
	MESH:C069880	PR:000003834	MESH:C566502
	CHEBI:45791	MESH:D007499	MESH:D013923
	MESH:C017899	SNP:RS3916967	SNP:RS3756648
	MESH:C005738	MESH:D001922	MESH:C116379
	MESH:C488736	MESH:C077114	MESH:C008885

Table S2. *Random Experiments*. HOC 2

Dataset	A Concept	B Concept	C Concept
HOC2	MESH:C040398	MESH:C069826	MESH:D018942
	MESH:C552268	PR:000002020	PR:000002096
	MESH:D008752	MESH:C051521	MESH:C525322
	PR:000024214	MESH:C119399	SNP:RS1800532
	MESH:C043576	PR:000013421	NCBITAXON:2214
	MESH:C108587	MESH:C536600	PR:000006048
	MESH:C565465	NCBITAXON:12280	MESH:C114206
	MESH:D000382	PR:000003284	MESH:D001819
	MESH:C420340	PR:000004507	MESH:D015095
	NCBITAXON:12118	MESH:C028304	MESH:C021341

Table S3. *Random Experiments*. HOC 3

Dataset	A Concept	B Concept	C Concept
HOC3	MESH:C520388	MESH:D003348	MESH:C510927
	MESH:C025417	MESH:D010190	CHEBI:8673
	MESH:C005388	PR:000007495	SNP:RS11669576
	MESH:C471771	MESH:C119418	MESH:C065753
	MESH:C488240	MESH:D014295	MESH:D017253
	MESH:C094195	OMIM:102530	PR:P22217
	PR:000006540	MESH:D060831	MESH:C085059
	SNP:RS5742612	MESH:C012036	MESH:C058700
	CHEBI:28902	MESH:C012391	MESH:D004108
	MESH:C454116	MESH:C494046	PR:000007201

Table S4. *Random Experiments*. HOC 4

Dataset	A Concept	B Concept	C Concept
HOC4	CHEBI:27119	MESH:D013392	MESH:C035179
	PR:O04348	MESH:D020721	PR:000003725
	MESH:C029699	PR:000009308	MESH:C538187
	PR:000006382	NCBITAXON:40085	MESH:C097135
	CHEBI:62064	CHEBI:23012	CHEBI:18042
	MESH:C114573	PR:P32854	PR:000005246
	MESH:C025182	MESH:C042413	MESH:C566157
	MESH:C535828	NCBITAXON:242718	PR:000016847
	MESH:C476143	MESH:C476748	MESH:C028033
	CHEBI:28518	NCBITAXON:10629	MESH:C053310

Table S5. *Random Experiments*. HOC 5

Dataset	A Concept	B Concept	C Concept
HOC5	CHEBI:59770	SNP:RS4072245	MESH:D008336
	MESH:C030669	MESH:C468590	CHEBI:49807
	NCBITAXON:48271	MESH:C040768	MESH:C562405
	SNP:RS4534195	SNP:RS2258447	SNP:RS4647693
	NCBITAXON:760192	PR:000012776	MESH:D014406
	NCBITAXON:39113	MESH:C094822	MESH:C008046
	MESH:C494537	MESH:C456047	PR:000009164
	MESH:C005939	MESH:C023386	PR:000004917
	MESH:C074335	MESH:C092901	SNP:RS996999
	MESH:C557508	MESH:D018817	NCBITAXON:37429
